# Carrying Angle Variation in Relation to Gender Among Children: An Observational Study

**DOI:** 10.31729/jnma.9016

**Published:** 2025-05-31

**Authors:** Sarbada Makaju, Pratima Palikhe, Sonam Chaudhary, Chandra Kala Rai

**Affiliations:** 1 Department of Anatomy, Kathmandu Medical College and Teaching Hospital, Duwakot, Bhaktapur, Nepal; 2 Department of Physiotherapy, Kathmandu Medical College and Teaching Hospital, Duwakot, Bhaktapur; 3 Department of Clinical Physiology, Maharajgunj Medical Campus, Institute of Medicine, Mahargunj, Kathmandu, Nepal; 4 Department of Physiology, Kathmandu Medical College and Teaching Hospital, Duwakot, Bhaktapur, Nepal

**Keywords:** *age*, *body mass index*, *carrying angle*

## Abstract

**Introduction:**

Carrying angle is the angle formed at the elbow joint during full extension and supination of arm and forearm which increases with increasing age till 14 years. This study measures the carrying angle variation with gender which can be beneficial for pediatricians in diagnosis, treatment planning of the related cases.

**Methods:**

The study was conducted 70 patients of Kathmandu Medical College and Teaching Hospital at Sinamangal and Duwakot between 15 December 2023 to 30 December 2023 after getting the ethical clearance from the Institutional Review Committee (reference no. KMC-IRC 08122023/02) with convenience sampling method. The carrying angle was measured by manual goniometer by drawing the axes in the arm and forearm. The data obtained was analyzed in different age groups and body mass index between boys and girls. The data was analyzed with Statistical Package for the Social Sciences version 25.

**Results:**

Out of 70 participants, 38(54.29%) were boys and 32(45.71%) were girls. The mean carrying angle among girls in right side was 10.53±2.52 degrees and 9.05±3.71 degrees for boys (p=0.06). Similarly, on left side it was 10.15±2.37 degrees for girls and 8.76±2.96 degrees for boys (p=0.036).

**Conclusions:**

The Carrying angle on both sides was found to be increased among female children in comparison to male irrespective of different age and BMI groups.

## INTRODUCTION

Carrying angle is the angle formed at the elbow joint when fully extended and supinated, between the long axes of the arm and forearm.^1^ It increases with age in children, which reaches its peak at around 14 years after which it starts to slightly decline and stabilized at the age of 16 years.^2,3^

The normal carrying angle is 5°-10° in males and 10°-15° in females; values above 15° suggest cubitus valgus, while those below 5° indicate cubitus varus.^4^ It is higher in females considered secondary sexual characteristic though some studies report no significant gender differences across age groups.^5-7^ Literatures have also showed BMI can influence the carrying angle.^8^

This study aims to evaluate normal range of carrying angle in children, focusing on gender-based variations across different age groups and BMI. These finding may assist pediatricians in early diagnosis, treatment planning, and surgical decisions for elbow-related conditions.

## METHODS

This descriptive study was carried out in the Department of Pediatric, Department of Pedodontics, Department of Orthopedic and Physiotherapy in Kathmandu Medical College and Teaching Hospital at Sinamangal and Duwakot from 15 December 2023 to 30 December 2023. A sample size (n) were calculated using formula of mean and standard deviation of carrying angle among 2 independent groups of males children was (11.12±3.8) and female children was (13.5±4.6) with the sample size formula of n = 2(Zα + Zβ) 2 × S.D 2 / (Mean1-Mean2)2 where, Zα=1.96; Zβ=0.84; S.D= Standard deviation (4.6); Mean 1=11.12; Mean 2= 13.5.^2^ Therefore, the total sample size calculated was 59 and the 15% additional data were collected in order to minimize the error. Hence, the total sample size in this study was taken 70.

In this study, the children between the age group of 6-15 years were included by convenience non-probability sampling.^2^ Any history of surgery, trauma and congenital disorder of upper limb present among the children were excluded.

The study was conducted after getting the ethical clearance from the Institutional review Committee of Kathmandu Medical College and Teaching Hospital (Refernce number: IRC 08122023/02). The children meeting the inclusion criteria were enrolled after taking informed consent. The carrying angle was measured by a manual goniometer by drawing the axes in the arm and forearm. The axis of the arm is defined by the lateral border of the superior surface of the acromion process to the midpoint of the lateral and medial epicondyles of the humerus in the full extension and supination position of upper limb. Another axis of the forearm was defined by the midpoint of the lateral and medial epicondyles of the humerus to the midpoint of the distal end of radial and ulnar styloid processes.^9,10^ Also, the height and weight were measured and the Body Mass Index (BMI) was calculated by dividing weight in kilograms by height in square meters. The Body Mass Index was evaluated as per their age as per WHO guidelines.^11,12^

The data collected was entered in Microsoft Office Excel worksheet and statistical analysis was done using Statistical Package for the Social Sciences (SPSS) version 25. Quantitative variables like age, gender and Body Mass index were computed for their distribution and mean and standard deviation as per the nature of data. This was further followed by mean of right and left carrying angles and also mean of carrying angles with various categories considered. P value of less than 0.05 was considered as significant.

## RESULTS

A total of number of 70 healthy children were participated in this study. The children between the ages of 6 to 10 years, were 41 (58.57%). Of the total participants, 38(54.29%) were boys and 32(45.71%) were girls. Additionally, 33 (47.14%) of the children had a normal Body Mass Index (BMI) for their age, according to WHO guidelines (Table 1).

**Table 1:** General Descriptives of the participants (n=70).

Variables	Frequency [n (%)]	Mean S.D
Age (years)		
6-10	41 (58.57%)	8.17 1.32
11-15	29 (41.43%)	12.44 1.18
Gender		
Boys	38 (54.29%)	
Girls	32 (45.71%)	
Body Mass Index (Kg/m2)		
Underweight	-	
Normal	33 (47.14%)	17.86 3.66
Overweight	13 (18.57%)	20.57 1.69
Obese	24 (34.29%)	25.17 4.65

In this study mean carrying angle of boys on right side was 9.05±3.71 degree and that of girls was 10.53±2.52 degree (p=0.06). Similarly, the mean carrying angle of boys on left side was 8.76±2.96 degree and that of girls was 10.15±2.37 degree (p=0.036), (Table 2).

**Table 2:** Distribution of carrying angles on right and left side in both the gender (n=70).

	Right		Left
Variables Range	Range	Mean SD	Range Mean SD
Gender			
Boys	3-19	9.053.71^*^	4-14 8.76 2.96^#^
Girls	6-16	10.532.52^*^	4-15 10.152.37^#^

Right=Right Carrying Angle (degree); Left= Left Carrying Angle (degree)

* p=0.06

# p=0.036

**Table 3:** Distribution of carrying angles on different independent variables (n=70).

Variables	Right		Left	
	Range	MeanSD	Range	MeanSD
Age				
6-10	3-19	9.483.38	4-15	9.142.98
11-15	4-18	10.063.17	4-14	9.752.45
BMI				
Normal	5-14	9.752.43	4-15	9.212.88
Above Normal	3-19	9.70 3.92	4-14	9.56 2.71

Right=Right Carrying Angle (degree); Left= Left Carrying Angle (degree)

The carrying angle on the right side for the age group 6-10 years was 9.48±.3.38 degrees and that for 1115 years was 10.06±3.17 degrees. Similarly, carrying angle on the right side for normal BMI was 9.75±2.43 degrees (Table 3).

The right carrying angle of male for the age group 6-10 years was 9.12±3.81 degrees and that for 11-15 years was 8.92±3.66 degrees (Table 4).

**Table 4:** Carrying angle in relation to the age and gender (n=70)

	Right		Left	
Age	Male (Mean S. D)	Female (Mean S. D)	Male (Mean S. D)	Female (Mean S. D)
6-10	9.123.81	10.002.69	8.453.13	10.112.54
11-15	8.923.66	11.132.26	9.282.67	10.202.24

Right=Right Carrying Angle (degree); Left= Left Carrying Angle (degree)

The right carrying angle of male with normal BMI was 9.50±3.20 degrees and that for female was 10.68±1.94 degrees (Table 5).

**Table 5:** Carrying angle in relation to the BMI and gender

	Right Carrying Angle (degree)		Left Carrying Angle (degree)	
BMI	Male (Mean S. D)	Female (Mean S. D)	Male (Mean S. D)	Female (Mean S. D)
Normal	9.503.20	10.681.94	8.953.08	10.002.30
Above normal	8.554.24	10.303.27	8.552.89	10.382.53

Right=Right Carrying Angle (degree); Left= Left Carrying Angle (degree)

## DISCUSSION

The carrying angle of the elbow joint is an important anatomical feature that refers to the angle formed between the long axis of the arm and long axis of forearm when the arm and forearm are fully extended and supinated in the position. The normal carrying angle is 5°-10° in males and 10°-15° in females.^4^ The carrying angle plays a key role in the functional mechanics of the upper limb like carrying the objects or swinging the arm during walking.

In this study, the carrying angle of the female children of the both sides were more than the male children, however it was statistically not significant for the right side. But the difference was statistically significant for left side. There are many studies conducted in the carrying angle in relation to the gender which claimed that the carrying angle was more in female than the male.^12,13^ The study also said that hormones may influence the Carrying angle in females. It may also be due to increased joint laxity in females permitting a greater degree of extension of elbow and hence greater Carrying angle in present in female. ^14^ Some studies have suggested that the dimensions of the female pelvis contribute to having the wider carrying angle in female. The broader carrying angle in females could also be linked to generally wider pelvis, which is associated with the anatomical positioning of the elbow.^15,16^ This larger carrying angle in females is thought to be an evolutionary adaptation related to childbirth, where the broader pelvis and altered limb alignment contribute to more effective balance and mobility.^16,17^ It is also said that even in the same gender the value of carrying angle decreases with increase in length of forearm and arm.^18^ In contrast, males typically exhibit narrower pelvic structures, which might explain their smaller carrying angle than in female.^5, 19^

In the present study, we observed that the right elbow exhibited a slightly larger carrying angle compared to the left. This trend was consistent across both genders, with both males and females showing a marginally greater carrying angle on the right side. This finding aligns with previous research, including studies conducted on the Andhra population, which also reported a larger carrying angle on the right side in both genders.^20^ The difference between the carrying angles of the right and left sides may suggest ligamentous flexibility at the elbow or asymmetrical bone growth and maturation of the humerus, the radius and ulna.^3,21^

It is said that age plays a crucial role in the development and maturation of the carrying angle. In our study, as the age increases, the carrying angle of the right and left hand of the female children were slightly increased. But in case of male children, the right side of the carrying angle wasn't increased as the age increased. In this study it observed overall a gradual increase in the carrying angle in the elbow joint with age group particularly during the pre-puberty and puberty stages. This study showed that the carrying angle increases as the child mature due to ossification, bone growth, changes in muscle tension and skeleton around the elbow joint. However, some researchers had also found that there is no significant difference in carrying angles in males and females of any age group.^6^ Moreover, several studies have demonstrated a gradual increase from childhood up to the age of 16 years. At this point, the carrying angle tends to stabilize. This stabilization of the carrying angle is important as it signifies the final stages of skeletal development^.3^ And the same results were found the study done by Golden et al.^22^

Regarding the impact of BMI on the carrying angle the findings are mixed. A study among adolescents in Abraka, Nigeria, observed a weak positive correlation between BMI and the left carrying angle suggesting that higher BMI might be associated with a slightly increased left carrying angle.^8^ However, in this study it showed slightly increased with carrying angle on the left side in female child with increased BMI. Additionally, a study conducted on obese females revealed a significant positive correlation between BMI and the carrying angle on both sides. The increased carrying angle observed in obese females could be attributed to the combined effect of increased in adipose tissue distribution around the joint and possible changes in bone morphology.^23^

Limitations of this study was measurement of carrying angles at a specific age, but does not allow for tracking individual changes over time. Moreover, the measurement techniques used in this study were based on manual goniometer which was non-invasive, cheaper and easy to perform in a single clinical setting. But they are often less precise and can be influenced by factors such as the examiner's skill, the child's cooperation and the position of the arm during measurement. This may lead to the measurement errors due to variations in positioning. This study would be stronger if the measurement was done in 3D imaging techniques or other advanced methods to capture the carrying angle more accurately and in dynamic movements. However, future similar studies in larger group sample can further support this evidence. Similarly, the sampling in the study was non probability which increases the biasness among the comparative groups which can be upgraded in future studies.

## CONCLUSION

This study concludes that the mean carrying angle on both sides was found to be increased among female children in comparison to male irrespective of different age and BMI groups considered. Hence, gender might also be considered as a prominent factor for the variation in carrying angle.
